# Supplementary material for: Genetic Evolution and Variation of Human Adenovirus Serotype 31 Epidemic Strains in Beijing, China, during 2010–2022
Source: Viruses. 2023 May 25;15(6):1240. doi: 10.3390/v15061240 (PMC10305296; doi:10.3390/v15061240)
Supplement: Supplementary file 1 [file viruses-15-01240-s001.zip › viruses-2393014-supplementary Figure S1.pdf]

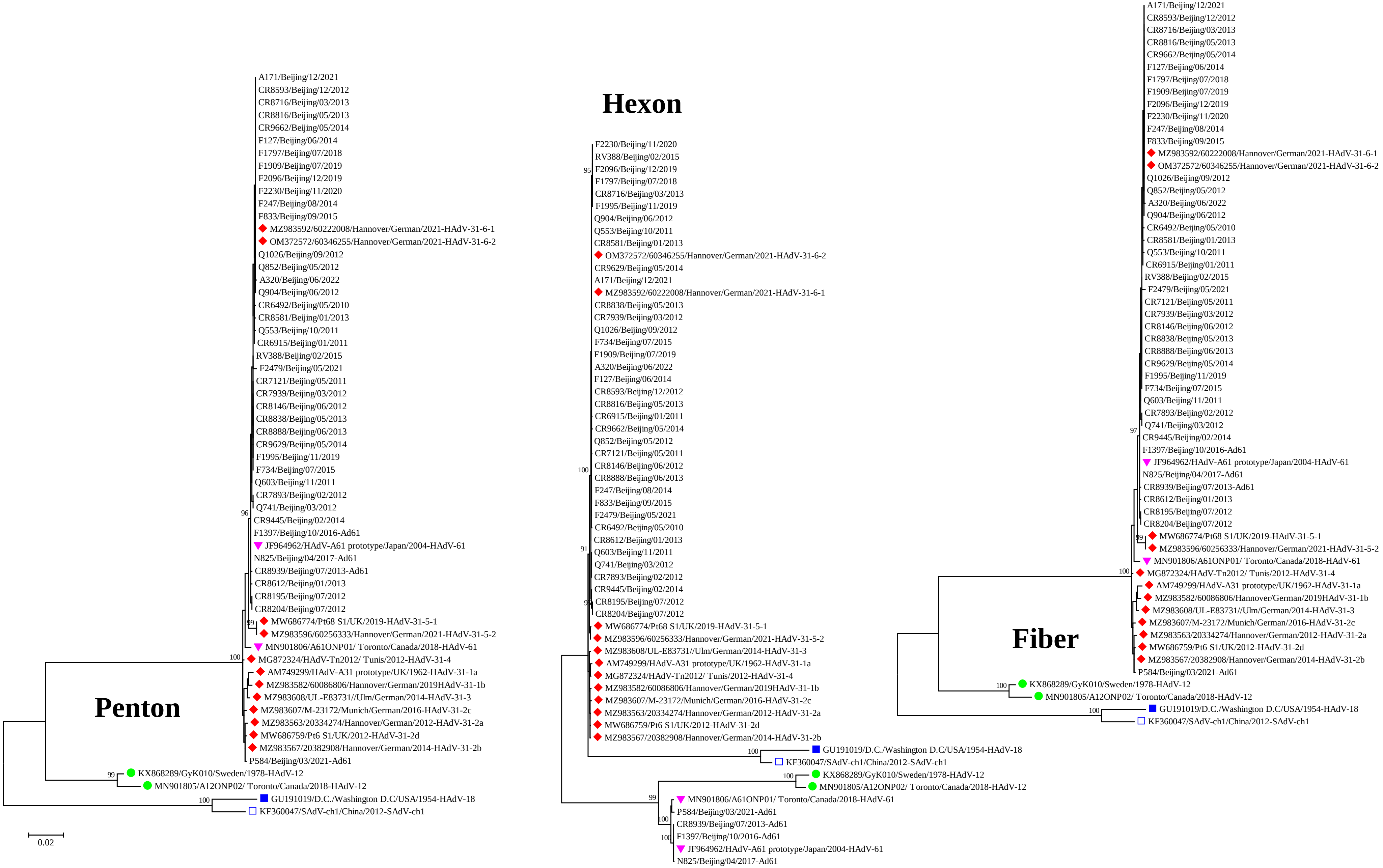
Penton
A171/Beijing/12/2021
CR8593/Beijing/12/2012
CR8716/Beijing/03/2013
CR8816/Beijing/05/2013
CR9662/Beijing/05/2014
F127/Beijing/06/2014
F1797/Beijing/07/2018
F1909/Beijing/07/2019
F2096/Beijing/12/2019
F2230/Beijing/11/2020
F247/Beijing/08/2014
F833/Beijing/09/2015
MZ983592/60222008/Hannover/German/2021-HAdV-31-6-1
OM372572/60346255/Hannover/German/2021-HAdV-31-6-2
Q1026/Beijing/09/2012
Q852/Beijing/05/2012
A320/Beijing/06/2022
Q904/Beijing/06/2012
CR6492/Beijing/05/2010
CR8581/Beijing/01/2013
Q553/Beijing/10/2011
CR6915/Beijing/01/2011
RV388/Beijing/02/2015
F2479/Beijing/05/2021
CR7121/Beijing/05/2011
CR7939/Beijing/03/2012
CR8146/Beijing/06/2012
CR8838/Beijing/05/2013
CR8888/Beijing/06/2013
CR9629/Beijing/05/2014
F1995/Beijing/11/2019
F734/Beijing/07/2015
Q603/Beijing/11/2011
CR7893/Beijing/02/2012
Q741/Beijing/03/2012
96
CR9445/Beijing/02/2014
F1397/Beijing/10/2016-Ad61
JF964962/HAdV-A61 prototype/Japan/2004-HAdV-61
N825/Beijing/04/2017-Ad61
CR8939/Beijing/07/2013-Ad61
CR8612/Beijing/01/2013
CR8195/Beijing/07/2012
CR8204/Beijing/07/2012
99
MW686774/Pt68 S1/UK/2019-HAdV-31-5-1
MZ983596/60256333/Hannover/German/2021-HAdV-31-5-2
MN901806/A61ONP01/ Toronto/Canada/2018-HAdV-61
100
MG872324/HAdV-Tn2012/ Tunis/2012-HAdV-31-4
AM749299/HAdV-A31 prototype/UK/1962-HAdV-31-1a
MZ983582/60086806/Hannover/German/2019HAdV-31-1b
MZ983608/UL-E83731//Ulm/German/2014-HAdV-31-3
MZ983607/M-23172/Munich/German/2016-HAdV-31-2c
MZ983563/20334274/Hannover/German/2012-HAdV-31-2a
MW686759/Pt6 S1/UK/2012-HAdV-31-2d
MZ983567/20382908/Hannover/German/2014-HAdV-31-2b
P584/Beijing/03/2021-Ad61
99
KX868289/GyK010/Sweden/1978-HAdV-12
MN901805/A12ONP02/ Toronto/Canada/2018-HAdV-12
100
GU191019/D.C./Washington D.C/USA/1954-HAdV-18
KF360047/SAdV-ch1/China/2012-SAdV-ch1
0.02
Hexon
F2230/Beijing/11/2020
RV388/Beijing/02/2015
95
F2096/Beijing/12/2019
F1797/Beijing/07/2018
CR8716/Beijing/03/2013
F1995/Beijing/11/2019
Q904/Beijing/06/2012
Q553/Beijing/10/2011
CR8581/Beijing/01/2013
OM372572/60346255/Hannover/German/2021-HAdV-31-6-2
CR9629/Beijing/05/2014
A171/Beijing/12/2021
MZ983592/60222008/Hannover/German/2021-HAdV-31-6-1
CR8838/Beijing/05/2013
CR7939/Beijing/03/2012
Q1026/Beijing/09/2012
F734/Beijing/07/2015
F1909/Beijing/07/2019
A320/Beijing/06/2022
F127/Beijing/06/2014
CR8593/Beijing/12/2012
CR8816/Beijing/05/2013
CR6915/Beijing/01/2011
CR9662/Beijing/05/2014
Q852/Beijing/05/2012
CR7121/Beijing/05/2011
100
CR8146/Beijing/06/2012
CR8888/Beijing/06/2013
F247/Beijing/08/2014
F833/Beijing/09/2015
F2479/Beijing/05/2021
CR6492/Beijing/05/2010
CR8612/Beijing/01/2013
91
Q603/Beijing/11/2011
Q741/Beijing/03/2012
CR7893/Beijing/02/2012
CR9445/Beijing/02/2014
CR8195/Beijing/07/2012
CR8204/Beijing/07/2012
MW686774/Pt68 S1/UK/2019-HAdV-31-5-1
MZ983596/60256333/Hannover/German/2021-HAdV-31-5-2
MZ983608/UL-E83731//Ulm/German/2014-HAdV-31-3
AM749299/HAdV-A31 prototype/UK/1962-HAdV-31-1a
MG872324/HAdV-Tn2012/ Tunis/2012-HAdV-31-4
MZ983582/60086806/Hannover/German/2019HAdV-31-1b
MZ983607/M-23172/Munich/German/2016-HAdV-31-2c
MZ983563/20334274/Hannover/German/2012-HAdV-31-2a
MW686759/Pt6 S1/UK/2012-HAdV-31-2d
MZ983567/20382908/Hannover/German/2014-HAdV-31-2b
100
GU191019/D.C./Washington D.C/USA/1954-HAdV-18
KF360047/SAdV-ch1/China/2012-SAdV-ch1
100
KX868289/GyK010/Sweden/1978-HAdV-12
MN901805/A12ONP02/ Toronto/Canada/2018-HAdV-12
99
MN901806/A61ONP01/ Toronto/Canada/2018-HAdV-61
P584/Beijing/03/2021-Ad61
100
CR8939/Beijing/07/2013-Ad61
100
F1397/Beijing/10/2016-Ad61
JF964962/HAdV-A61 prototype/Japan/2004-HAdV-61
N825/Beijing/04/2017-Ad61
Fiber
A171/Beijing/12/2021
CR8593/Beijing/12/2012
CR8716/Beijing/03/2013
CR8816/Beijing/05/2013
CR9662/Beijing/05/2014
F127/Beijing/06/2014
F1797/Beijing/07/2018
F1909/Beijing/07/2019
F2096/Beijing/12/2019
F2230/Beijing/11/2020
F247/Beijing/08/2014
F833/Beijing/09/2015
MZ983592/60222008/Hannover/German/2021-HAdV-31-6-1
OM372572/60346255/Hannover/German/2021-HAdV-31-6-2
Q1026/Beijing/09/2012
Q852/Beijing/05/2012
A320/Beijing/06/2022
Q904/Beijing/06/2012
CR6492/Beijing/05/2010
CR8581/Beijing/01/2013
Q553/Beijing/10/2011
CR6915/Beijing/01/2011
RV388/Beijing/02/2015
F2479/Beijing/05/2021
CR7121/Beijing/05/2011
CR7939/Beijing/03/2012
CR8146/Beijing/06/2012
CR8838/Beijing/05/2013
CR8888/Beijing/06/2013
CR9629/Beijing/05/2014
F1995/Beijing/11/2019
F734/Beijing/07/2015
Q603/Beijing/11/2011
CR7893/Beijing/02/2012
Q741/Beijing/03/2012
97
CR9445/Beijing/02/2014
F1397/Beijing/10/2016-Ad61
JF964962/HAdV-A61 prototype/Japan/2004-HAdV-61
N825/Beijing/04/2017-Ad61
CR8939/Beijing/07/2013-Ad61
CR8612/Beijing/01/2013
CR8195/Beijing/07/2012
CR8204/Beijing/07/2012
99
MW686774/Pt68 S1/UK/2019-HAdV-31-5-1
MZ983596/60256333/Hannover/German/2021-HAdV-31-5-2
MN901806/A61ONP01/ Toronto/Canada/2018-HAdV-61
100
MG872324/HAdV-Tn2012/ Tunis/2012-HAdV-31-4
AM749299/HAdV-A31 prototype/UK/1962-HAdV-31-1a
MZ983582/60086806/Hannover/German/2019HAdV-31-1b
MZ983608/UL-E83731//Ulm/German/2014-HAdV-31-3
MZ983607/M-23172/Munich/German/2016-HAdV-31-2c
MZ983563/20334274/Hannover/German/2012-HAdV-31-2a
MW686759/Pt6 S1/UK/2012-HAdV-31-2d
MZ983567/20382908/Hannover/German/2014-HAdV-31-2b
P584/Beijing/03/2021-Ad61
100
KX868289/GyK010/Sweden/1978-HAdV-12
MN901805/A12ONP02/ Toronto/Canada/2018-HAdV-12
100
GU191019/D.C./Washington D.C/USA/1954-HAdV-18
KF360047/SAdV-ch1/China/2012-SAdV-ch1
